# Chemical Dissection of the Link between Streptozotocin, *O*-GlcNAc, and Pancreatic Cell Death

**DOI:** 10.1016/j.chembiol.2008.06.010

**Published:** 2008-08-25

**Authors:** Shalini Pathak, Helge C. Dorfmueller, Vladimir S. Borodkin, Daan M.F. van Aalten

**Affiliations:** 1 Division of Biological Chemistry and Drug Discovery, College of Life Sciences, University of Dundee, Dundee DD1 5EH, Scotland

**Keywords:** CHEMBIO, SIGNALING

## Abstract

Streptozotocin is a natural product that selectively kills insulin-secreting β cells, and is widely used to generate mouse models of diabetes or treat pancreatic tumors. Several studies suggest that streptozotocin toxicity stems from its *N*-nitrosourea moiety releasing nitric oxide and possessing DNA alkylating activity. However, it has also been proposed that streptozotocin induces apoptosis by inhibiting *O*-GlcNAcase, an enzyme that, together with *O*-GlcNAc transferase, is important for dynamic intracellular protein *O*-glycosylation. We have used *galacto*-streptozotocin to chemically dissect the link between *O*-GlcNAcase inhibition and apoptosis. Using X-ray crystallography, enzymology, and cell biological studies on an insulinoma cell line, we show that, whereas streptozotocin competitively inhibits *O*-GlcNAcase and induces apoptosis, its *galacto*-configured derivative no longer inhibits *O*-GlcNAcase, yet still induces apoptosis. This supports a general chemical poison mode of action for streptozotocin, suggesting the need for using more specific inhibitors to study protein *O*-GlcNAcylation.

## Introduction

Streptozotocin (STZ) is an *N*-methyl-*N*-nitrosoureido D-glucosamine derivative originally isolated from *Streptomyces achromogenes* half a century ago (Vavra et al., 1959). It was subsequently discovered to be particularly toxic to pancreatic β cells that secrete insulin, and has since been used extensively to create animal models of type I diabetes (Mansford and Opie, 1968). Due to its selective toxicity, it is now also in use for the treatment of cancer of the pancreatic islets (Brentjens and Saltz, 2001).

Despite its use for over several decades, the mode of action of STZ is not fully understood. Two, essentially conflicting, mechanisms have been proposed. The first (termed the “chemical poison model” here) is linked to the *N*-nitrosourea group that STZ carries (see below for STZ chemical structure). Through chemical decomposition, this group can act as an alkylating agent and/or a nitric oxide (NO) donor. Biochemical evidence has suggested that STZ does indeed increase NO in pancreatic β cells (Turk et al., 1993; Kaneto et al., 1995). Extensive literature also supports a genotoxic effect of STZ through its ability to directly alkylate DNA (Yamamoto et al., 1981; Bolzán and Bianchi, 2002)—indeed, it is a general antibiotic with mutagenic activity on both bacterial and eukaryotic cells (Gichner et al., 1968; Bolzán and Bianchi, 2002). It was initially unclear how STZ was selectively toxic against β cells, but recent overexpression studies and experiments with colorimetric STZ derivatives have demonstrated that GLUT2, a glucose transporter selectively expressed in pancreatic islets, also transports STZ (Schnedl et al., 1994; Ran et al., 2007).

An entirely different explanation of STZ toxicity was proposed more recently, and is based on the link between STZ and a cytosolic protein posttranslational modification through *O*-glycosylation with *N*-acetylglucosamine (*O*-GlcNAc), referred to here as the “*O*-GlcNAc-dependent model” of STZ toxicity. Protein *O*-GlcNAcylation was discovered two decades ago, and is an abundant, dynamic, and inducible posttranslational modification of serine/threonine residues on intracellular proteins in higher eukaryotes (Zachara and Hart, 2006; Hart et al., 2007). *O*-GlcNAcylation has been shown to be involved in diverse cellular processes such as the cell cycle, nutrient sensing, stress response, proteasomal regulation, and the response to insulin. Two proteins, conserved from *Caenorhabditis elegans* to human, are involved in maintaining *O*-GlcNAc levels. The *O*-GlcNAc transferase (OGT) transfers GlcNAc from the sugar nucleotide UDP-GlcNAc to acceptor serines/threonines on proteins (Haltiwanger et al., 1992; Hart et al., 2007). The *O*-GlcNAcase (OGA) hydrolyzes *O*-GlcNAcylated proteins to the free protein and GlcNAc (Gao et al., 2001; Hart et al., 2007). Several reports have suggested that STZ kills pancreatic β cells in an *O*-GlcNAc-dependent manner, based on a number of observations. First, it was observed that millimolar concentrations of STZ were able to raise general levels of protein *O*-GlcNAcylation and that OGT was particularly abundant in the pancreas—rapidly leading to protein hyper-*O*-GlcNAcylation under conditions of OGA inhibition (Lubas et al., 1997; Roos et al., 1998; Hanover et al., 1999; Akimoto et al., 1999). It was then noted that STZ is a GlcNAc derivative and was found to inhibit OGA in the millimolar range (Roos et al., 1998; Hanover et al., 1999; Liu et al., 2000; Konrad et al., 2001). The mode of inhibition was proposed to involve covalent modification of the enzyme or the enzyme-catalyzed formation of a tight binding inhibitor (Konrad et al., 2001; Toleman et al., 2006). Putting these data together, it has been proposed that STZ specifically kills islet cells by inhibiting OGA, resulting in hyper-*O*-GlcNAcylation and activation of stress pathways leading to apoptosis (Liu et al., 2000; Konrad et al., 2001).

The cell biological implications of *O*-GlcNAc have attracted rapidly increasing interest over the last few years (Zachara and Hart, 2006; Hart et al., 2007). In particular, the possible competition between *O*-GlcNAcylation and phosphorylation (OGT/kinases targeting the same serines/threonines) has become a topic of vigorous research, due to the possibility of *O*-GlcNAc regulating a number of phosphorylation-dependent signal transduction pathways. Many studies have used knockout/knockdown/overexpression approaches to modulate levels of the OGA/OGT proteins, thus testing the effects of hyper/hypo-*O*-GlcNAcylation on particular cellular processes (e.g., Slawson et al., 2005; Hu et al., 2006; Yang et al., 2008). However, an alternative approach has been to use small-molecule inhibitors on live cells to inhibit OGA, inducing hyper-*O*-GlcNAcylation (potent inhibitors of OGT are not yet available). STZ and PUGNAc (Haltiwanger et al., 1998), a nanomolar, but nonspecific, OGA inhibitor, have been extensively used for such studies in the past decade, although more potent/selective inhibitors (thiazoline derivatives [Whitworth et al., 2007] and GlcNAcstatin [Dorfmueller et al., 2006]) have recently become available. For instance, STZ has been used to study the link between *O*-GlcNAc and p53 degradation (Yang et al., 2006), effects of *O*-GlcNAc levels on the insulin signaling pathway (Matthews et al., 2007), and *O*-GlcNAc-dependent regulation of the proteasome (Liu et al., 2004). However, as long as the mode of STZ action has not been established, the value of such studies is uncertain.

Interestingly, two reports have recently called into question the hypothesis that STZ kills β cells in an *O*-GlcNAc-dependent manner. Comparing the STZ and (the more potent) PUGNAc inhibitor, it was noted that although both inhibitors similarly raised *O*-GlcNAc levels, only STZ induced apoptotic DNA fragmentation and decreased insulin secretion and protein synthesis, resulting in cell death (Gao et al., 2000). Similarly, whereas overexpression of a key enzyme in the UDP-GlcNAc biosynthetic pathway led to the expected increase in *O*-GlcNAc levels, oxidative stress, and β-cell-specific protein expression levels, these effects could not be reproduced with PUGNAc (Kaneto et al., 2001).

Here we have used an alternative chemical approach to further distinguish between the “chemical poison” and “*O*-GlcNAc-dependent” modes of STZ action. Capitalizing on available structural data, a *galacto*-configured isomer of STZ was designed that no longer inhibits OGA but is still imported into pancreatic cells. We show that this STZ isomer is equally potent as STZ in inducing programmed DNA fragmentation, activation of caspase 3, and increasing membrane phosphatidylserine levels, all hallmarks of apoptosis. These data support the chemical poison mode of STZ action, where DNA damage leads to programmed cell death, and establish that STZ does not kill β cells in an *O*-GlcNAc-dependent manner.

## Results and Discussion

### Structure of the STZ-OGA Complex

**Figure 1:**
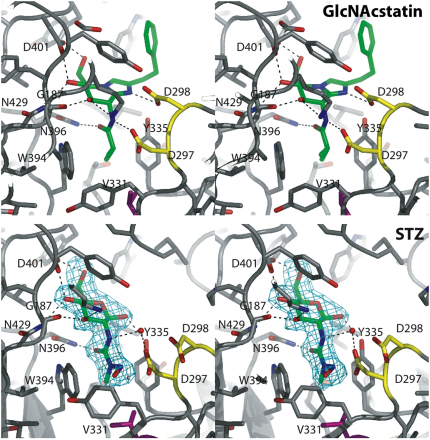
Structure of the *Cp*OGA-STZ Complex Stereo figures of the crystallographically determined complexes of *Cp*OGA with GlcNAcstatin (Protein Data Bank ID code 2J62; Dorfmueller et al., 2006) and STZ. The *Cp*OGA structure is shown as a gray ribbon, with active site residues shown as sticks with gray carbon atoms, except for two key catalytic residues (Asp297/Asp298, yellow) and the active site Val331 (magenta). The inhibitors are shown as stick models with green carbon atoms. An ordered water molecule (red sphere) is shown in the STZ structure. Hydrogen bonds are indicated by black dashed lines. An unbiased |F_o_| − |F_c_|, ϕ_calc_ electron density map (3.0 σ) for STZ is shown in cyan.

Previous reports have suggested that streptozotocin is a weak (mM) inhibitor of human OGA (hOGA), acting through a covalent suicide mechanism (Konrad et al., 2001). A very recent NMR study has suggested a rearrangement of the nitrosourea STZ side chain, resulting in the formation of an oxazoline-bearing structure that was proposed to be a tight-binding inhibitor (Toleman et al., 2006). To investigate this in more detail, we studied binding of STZ to a bacterial OGA, the *Clostridium perfringens* OGA (*Cp*OGA; Rao et al., 2006), using X-ray crystallography. *Cp*OGA possesses an active site nearly identical to hOGA and binds substrates/inhibitors with similar affinities (Rao et al., 2006; Dorfmueller et al., 2006). Diffraction data of the *Cp*OGA-STZ complex were obtained to 2.2 Å and the structure was refined to a model with an R factor of 0.196 (R_free_ = 0.241). Early on in the refinement, well-defined |F_o_| − |F_c_|, ϕ_calc_ density was observed for STZ in the *Cp*OGA active site (Figure 1). The STZ pyranose ring occupies a position similar to that of the potent OGA inhibitor GlcNAcstatin (maximum positional shift = 1.0 Å; Figure 1). The pyranose ring assumes a _4_C^1^ conformation and is in the β configuration. The nitrosourea moiety points toward the bottom of the active site, occupying a position similar to that of the isobutanamide group of GlcNAcstatin (Figure 1). Many of the interactions observed in the *Cp*OGA-GlcNAcstatin complex are also present in the STZ complex, involving residues Asn396, Asn429, and Asp401. Strikingly, however, no interactions are seen between STZ and the catalytic machinery (Asp297/Asp298) that tightly engages GlcNAcstatin in the *Cp*OGA-GlcNAcstatin complex (Figure 1). The overall conformation of the protein in the *Cp*OGA-STZ complex is more similar to the apo *Cp*OGA structure (root-mean-square deviation [rmsd] on Cα atoms = 0.3 Å) than to the GlcNAcstatin complex (rmsd = 1.0 Å). The electron density does not support a covalent interaction between protein and inhibitor, or the presence of an oxazolinium ion generated by the rearrangement of the nitrosourea group, as proposed recently (Toleman et al., 2006).

### STZ Is a Competitive, but Not Suicidal, OGA Inhibitor

**Figure 2:**
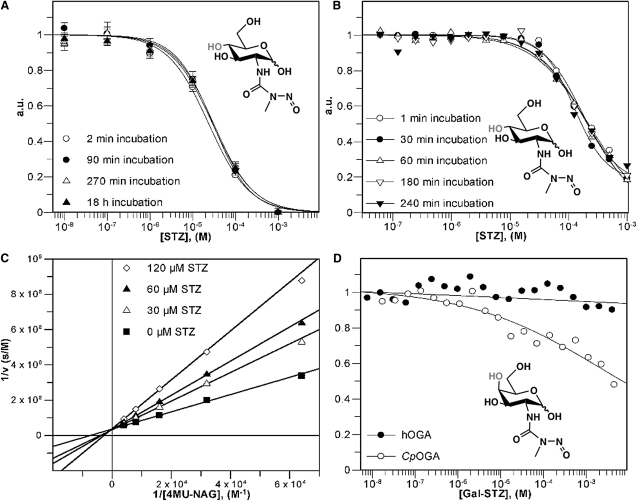
*Cp*OGA and *h*OGA Inhibition by STZ and Gal-STZ (A and B) Dose-response curves of streptozotocin (STZ, chemical structure shown in inset), preincubated with *Cp*OGA or hOGA for different lengths of time prior to the start of the reaction. Data were fitted using the standard IC_50_ equation in the GraFit program (Leatherbarrow, 2001). (C) Lineweaver-Burk analysis of hOGA steady-state kinetics measured in the presence of 0–120 μM STZ, preincubated with the inhibitor for 3 min. Data were fitted using the standard equation for competitive inhibition in the GraFit program (Leatherbarrow, 2001). (D) Dose-response curves of *galacto*-configured streptozotocin (Gal-STZ, chemical structure shown in inset), against hOGA and *Cp*OGA. The curves shown represent approximate fits using the standard IC_50_ equation in the GraFit program (Leatherbarrow, 2001). Accurate fits could not be obtained due to the weak inhibitory activity of Gal-STZ, and the IC_50_s are taken to be >100 mM for hOGA and >10 mM for *Cp*OGA.

To further investigate the mode of inhibition of STZ, *Cp*OGA activity was measured using the 4-methylumbelliferone-GlcNAc assay with a range of STZ concentrations and varying preincubation times of enzyme and inhibitor (2 min–18 hr; Figure 2A). Both commercial (Sigma) and resynthesized samples showed that STZ inhibits *Cp*OGA with an IC_50_ of 30 μM (Figure 2A). However, the dose-response curves did not show an incubation time-dependent shift, suggesting STZ does not inhibit OGA through a covalent mechanism, in agreement with the structural data.

*Cp*OGA contains an active site that is nearly identical to that of hOGA (Rao et al., 2006). However, a notable difference is *Cp*OGA Val331, which is a cysteine (Cys215) in hOGA. hOGA has been reported to be sensitive to thiol-reactive compounds (Dong and Hart, 1994), and the involvement of cysteines in STZ inactivation of hOGA has been proposed (Lee et al., 2006). Thus, we also studied the time dependence of STZ hOGA inhibition. hOGA (K_m_ = 80 ± 6 μM, k_cat_ = 13.9 ± 0.5 s^−1^) showed similar steady-state kinetics as wild-type *Cp*OGA (K_m_ = 4.5 ± 1.7 μM, k_cat_ = 6.0 ± 0.8 s^−1^), in agreement with the conserved active sites. Steady-state kinetics were measured at different substrate/STZ concentrations with 1–240 min preincubation of the enzyme/inhibitor mixture (Figures 2B and 2C). STZ inhibited hOGA competitively with K_i_ = 64 ± 3 μM with 3 min incubation, and there is no change in the dose-response curve for the longer time periods of incubation. Thus, in agreement with the structural data that show free, intact STZ binding to the OGA active site, there is no evidence for a time-dependent suicide/covalent inhibitory mechanism.

### *Galacto*-Configured STZ Is a Poor OGA Inhibitor

Several studies have proposed that the pancreatic β cell toxicity of STZ is due to its ability to inhibit β cell OGA, increasing general levels of protein *O*-GlcNAcylation and driving cells toward apoptosis (Liu et al., 2000; Konrad et al., 2001). We decided to investigate this further with the help of a chemical probe. Early kinetic characterization of OGA has shown that the enzyme is inhibited by GlcNAc, but not by GalNAc, unlike the GH 20 lysosomal hexosaminidases (Gao et al., 2001). This is readily explained by the structural data, which show that a conserved aspartic acid (Asp401 in *Cp*OGA, Asp485 in hOGA) tethers the O6 and equatorial O4 hydroxyls (Figure 1). Indeed, mutation of Asp401 to an alanine abrogates *Cp*OGA activity (Rao et al., 2006). Thus, we anticipated that a *galacto*-configured STZ analog (Gal-STZ; Figure 2D) would no longer be an OGA inhibitor, while maintaining the reactive nitrosourea group. This would be a useful tool to dissect the *O*-GlcNAc-dependent and the chemical poison models of pancreatic β cell toxicity of STZ.

Gal-STZ was synthesized from D-galactosamine and *N*-methyl-*N*-nitrosocarbamic acid *N*′-hydroxysuccinimide ester to warrant the regioselective positioning of the *N*-nitroso group as previously described for STZ (Martinez et al., 1982). A dose-response curve for Gal-STZ against hOGA reveals that Gal-STZ inhibits the human enzyme with IC_50_s > 100 mM (Figure 2D). This is at least three orders of magnitude weaker than what was measured for STZ (Figure 2A). Thus, Gal-STZ is a useful tool to study possible differences in effects on pancreatic β cells compared to STZ.

### STZ, but Not Gal-STZ, Raises Cellular *O*-GlcNAc Levels

**Figure 3:**
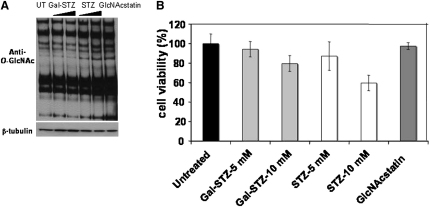
STZ-Induced *O*-GlcNAcylation and Min6 Cell Viability (A) Western blot of Min6 insulinoma cell lysates probed for *O*-GlcNAcylated proteins with an anti-*O*-GlcNAc antibody (CTD110.6). Cells were treated with 5/10 mM STZ or Gal-STZ or 20 μM GlcNAcstatin for 16 hr. (B) Trypan blue cell viability assay of Min6 cells (24-well plate, 1 × 10^5^ cells/well) treated with STZ or Gal-STZ (5 mM or 10 mM) or GlcNAcstatin (20 μM) for 6 hr. The live cells were measured in triplicate and untreated cells were taken as 100% viable. Data errors represent the standard deviation of the mean.

Incubation with STZ has been shown to raise general *O*-GlcNAc levels on cytosolic proteins (Konrad et al., 2001; Liu et al., 2000). In our hands, incubation of Min6 insulinoma cells with high (5–10 mM) concentrations of STZ leads to an observable increase in general *O*-GlcNAcylation as qualitatively assessed from an anti-*O*-GlcNAc western blot (Figure 3A). By comparison, the potent, and selective, OGA inhibitor GlcNAcstatin shows significantly larger increases in *O*-GlcNAcylation when incubated in micromolar concentrations with Min6 cells (Figure 3A). Gal-STZ, used at the same concentrations as STZ, does not induce observable changes in *O*-GlcNAcylation, in agreement with the enzyme inhibition data.

### Both STZ and Gal-STZ, but Not GlcNAcstatin, Reduce Insulinoma Cell Viability

STZ is known to cause pancreatic β cell death, and a number of, sometimes conflicting, mechanisms have been proposed to explain this. A large body of data supports a chemical mechanism where the nitrosourea group acts as a nitric oxide donor and/or alkylating agent, essentially poisoning the cell (Turk et al., 1993; Kaneto et al., 1995; Yamamoto et al., 1981; Bolzán and Bianchi, 2002). An alternative mechanism is cell death induced by hyper-*O*-GlcNAcylation, through inhibition of OGA. Gal-STZ is a precise chemical tool to distinguish these mechanisms—it is almost isosteric to STZ and possesses the nitrosourea group, yet does not inhibit OGA. We examined Min6 cell viability in the presence of 5–10 mM STZ or Gal-STZ (Figure 3B). Both compounds significantly decreased cell viability, to (within experimental error) similar levels. Interestingly, however, the potent OGA inhibitor GlcNAcstatin does not affect cell viability, despite its ability to generate larger increases in general *O*-GlcNAcylation (Figures 3A and 3B).

### Both STZ and Gal-STZ, but Not GlcNAcstatin, Induce Apoptosis

**Figure 4:**
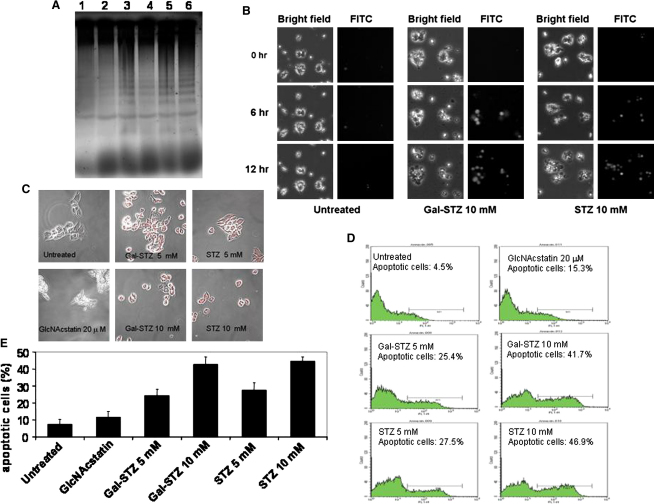
STZ/Gal-STZ-Induced Apoptosis in Min6 Cells (A) DNA fragmentation assay of untreated Min6 cells and cells treated with Gal-STZ, STZ, or GlcNAcstatin for 6 hr. Lane 1: untreated cells; lane 2: GlcNAcstatin (20 μM); lanes 3 and 4: Gal-STZ (5/10 mM); lanes 5 and 6: STZ (5/10 mM). (B) Min6 cells stained for caspase 3 activity. Cells were grown on microslides. After adding Gal-STZ, STZ, or GlcNAcstatin and the NucView caspase substrate, the slides were set up in the incubation chamber at 37°C and cells were imaged with a Leica inverted microscope every hour for 12 hr. NucView fluorescence signal was detected using a FITC filter set. The scale bar represents 130 μm. (C) Confocal microscopy images showing activated caspase 3 using a caspase 3 (Asp175) antibody (red) in Min6 cells treated with Gal-STZ, STZ, or GlcNAcstatin. After fixation, cells were incubated with primary antibody, followed by Texas red-conjugated secondary antibody, and visualized. (D and E) Early apoptosis as detected with Annexin V-FITC labeling of the cells. Min6 cells were treated with Gal-STZ, STZ, or GlcNAcstatin for 16 hr. After trypsinization, cells (3–4 × 10^5^) were resuspended in Annexin V buffer and incubated with Annexin V for 5 min before analyzing Annexin V binding by flow cytometry. Gal-STZ and STZ showed more than 40% cell death at 10 mM concentration. (D) A representative example of flow cytometric analysis. (E) A histogram showing data representing mean ± SD in triplicate cultures.

To investigate the mechanism of STZ/Gal-STZ-induced cell death, we attempted to distinguish between necrosis and apoptosis using a number of approaches. One of the hallmarks of apoptosis is controlled DNA fragmentation (Duke et al., 1983). Compared to a healthy control population of Min6 cells, cells treated with 5–10 mM Gal-STZ or STZ showed significant, and similar, levels of DNA fragmentation (Figure 4A). Qualitatively, no such increases in DNA fragmentation were observed for GlcNAcstatin. Similar results were obtained when drug-treated and control Min6 cell populations were investigated under the microscope. Caspase 3 is part of the caspase cascade activated in apoptosis. Both STZ and Gal-STZ, but not GlcNAcstatin, induce processing of full-length caspase down to the active protease fragment, as observed in fixed Min6 cells using an anti-caspase 3 antibody specific for the processed, active, form (Figure 4B). Similar results were obtained when caspase 3 activity was investigated in live cells, using a fluorescent caspase 3 substrate. During 12 hr incubation with either STZ or Gal-STZ, caspase 3 activity increases and reaches similar levels (Figure 4C). To quantitatively measure activation of the controlled cell death program, we studied levels of phosphatidylserine displayed on the Min6 cell surface. A fluorescence-assisted cell sorting (FACS) approach was used with FITC-labeled Annexin V (Figures 4D and 4E). Compared to control cells and a GlcNAcstatin-treated population, STZ/Gal-STZ treatment significantly increased the fraction of cells displaying high concentrations of phosphatidylserine on the membrane.

### Concluding Remarks

The mechanism through which STZ selectively kills the insulin-secreting β cells in the pancreas has been the subject of a wide range of studies. Genetic and biochemical approaches have been used to propose that the source of its toxicity lies in the *N*-nitrosourea moiety, acting as a source of nitric oxide and/or an alkyl donor (Turk et al., 1993; Kaneto et al., 1995; Yamamoto et al., 1981; Bolzán and Bianchi, 2002). The enigma of why this would be specific to β cells was solved by elegant overexpression/genetic studies that demonstrated that GLUT2 is specifically expressed in the pancreas and is the only glucose transporter that recognizes and translocates STZ (Schnedl et al., 1994; Ran et al., 2007). This would reduce the STZ glucosamine sugar to simply a transport mechanism for getting the reactive *N*-nitrosourea moiety into the cell. However, it was also noted that STZ is a GlcNAc derivative and, when tested on OGA, indeed revealed OGA inhibitory activity (Konrad et al., 2001). It was also noted that the pancreas contained unusually high concentrations of OGT, whereas parallel studies demonstrated that high levels of OGT/*O*-GlcNAc could drive cells toward apoptosis (Liu et al., 2000, 2004; Slawson et al., 2005). Studies with insulinoma cell lines and streptozotocin then showed a correlation between increased protein *O*-GlcNAcylation (inhibition of OGA will lead to unbalanced OGT activity) and cell death (Konrad et al., 2001). Following on from this work, research on the role of *O*-GlcNAc in many different cellular processes has included the use of streptozotocin as an agent to modulate *O*-GlcNAc levels in cells (e.g., Liu et al., 2004; Matthews et al., 2007; Yang et al., 2006), despite uncertainty concerning its mode of action.

Earlier work had already demonstrated that PUGNAc, a much more potent OGA inhibitor than STZ, although able to significantly increase *O*-GlcNAc levels did not induce β cell death, arguing against a link between STZ, *O*-GlcNAc, and apoptosis (Gao et al., 2000; Kaneto et al., 2001). We sought to further investigate this link using an alternative chemical approach. Although our structure of the *Cp*OGA-STZ complex does not support formation of a covalent intermediate or a tight-binding oxazolinium ion as recently suggested (Toleman et al., 2006), this complex was obtained by soaking procedures and cannot be taken as conclusive proof of the absence of such mechanisms. Nevertheless, in our hands, STZ, either resynthesized or from a commercial source, is a weak but purely competitive inhibitor without any evidence of time-dependent inhibition. Inspired by the *Cp*OGA-STZ complex, the *galacto* isomer of STZ (Gal-STZ) was synthesized. This compound, as expected, no longer inhibits OGA. This also extended to cellular studies, where STZ was able to disrupt the balance between *O*-GlcNAc transfer and hydrolysis, whereas no such effect was observed for Gal-STZ. Crucially, however, STZ and Gal-STZ were equally able to induce apoptosis in the Min6 insulinoma cell line, as evidenced by total cell viability, induction of caspase 3 activity, and phosphatidylserine levels on the cell surface (Figures 3 and 4). Furthermore, the picomolar OGA inhibitor GlcNAcstatin, although able to raise *O*-GlcNAc levels, also does not induce apoptosis. This is in line with earlier reports showing that the OGA inhibitor PUGNAc (six orders of magnitude more potent than STZ) does not induce cell death (Gao et al., 2000; Kaneto et al., 2001; Okuyama and Yachi, 2001). In support of these earlier studies, the data reported here uncouple the ability of STZ to induce apoptosis from its activity as an OGA inhibitor.

The discovery of *O*-GlcNAc more than two decades ago has given rise to significant research activity to discover how this posttranslational modification might regulate cellular processes, in particular through interplay with protein phosphorylation (Zachara and Hart, 2006; Hart et al., 2007). Although overexpression/gene knockout strategies have been pursued, interpretation of their results are complicated by the fact that both OGA and OGT are known to participate in multiprotein complexes. Chemical intervention with small-molecule inhibitors is a possible alternative, although it is crucial to ensure the agents used are selective. The work described here shows that STZ, now a widely used inhibitor to study protein *O*-GlcNAcylation, kills β cells in an *O*-GlcNAc-independent manner, supporting instead the chemical poison mode of action. It will therefore be more appropriate to use more potent, and selective inhibitors, of OGA, such as PUGNAc (Haltiwanger et al., 1998), GlcNAcstatin (Dorfmueller et al., 2006), and the thiazolines (Whitworth et al., 2007) for cell biological studies into the role of *O*-GlcNAc.

## Significance

**Streptozotocin (STZ) is widely used to generate mouse models of diabetes, or to treat pancreatic tumours. It has been proposed that STZ toxicity is caused by its ability to inhibit *O*-GlcNAcse, thereby raising levels of the intracellular *O*-GlcNAc modification to lethal levels. This work attempts to further study the mode of action of this drug by studying *galacto*-configured isomer of STZ. We show that while streptozotocin competitively inhibits *O*-GlcNAcase and induces apoptosis, its galacto-configured derivative no longer inhibits *O*-GlcNAcase, yet still induces apoptosis. This novel chemical tool strengthens the notion that STZ is not a specific inhibitor of *O*-GlcNAcase but rather a general cytotoxic compound.**

## Experimental Procedures

### Mutagenesis, Protein Expression, and Purification

Wild-type *Cp*OGA was expressed and purified as described previously (Rao et al., 2006). The purified protein was concentrated to 15 mg/ml and diluted to the desired concentration in buffer (50 mM citric acid, 125 mM NaH_2_PO_4_ [pH 5.5]).

A truncated form of hOGA (residues 53–916) was cloned and expressed as a GST fusion in *Escherichia coli* as described elsewhere (H.C.D. and D.M.F.v.A., unpublished results). The purified GST-hOGA protein was dialyzed into 50 mM Tris-HCl (pH 7.5), 0.1 mM EGTA, 150 mM NaCl_2_, 0.07% β-mercaptoethanol, 0.1 mM PMSF, 1 mM benzamidine.

### Enzymology

Enzyme assays were carried out as described previously (Rao et al., 2006; Dorfmueller et al., 2006). STZ and Gal-STZ were dissolved to a concentration of 100 mM in water. Steady-state kinetics of *Cp*OGA and hOGA were determined using the fluorogenic substrate 4-methylumbelliferyl-*N*-acetyl-β-D-glucosaminide (4MU-GlcNAc; Sigma). Standard reaction mixtures (50 μl) contained 0.2 nM *Cp*OGA or 2 nM hOGA in McIlvaine buffer (0.2 M Na_2_HPO_4_ mixed with 0.1 M citric acid to pH 6.8) supplemented with 0.1 mg/ml BSA, and 0–250 μM of substrate in water. The reaction was run at room temperature for 7 min (*Cp*OGA) or 60 min (hOGA). The reaction was stopped by the addition of 100 μl of 3 M glycine-NaOH (pH 10.3). The fluorescence of the released 4-methylumbelliferone (4MU) was quantified using a FLX 800 microplate fluorescence reader (Bio-Tek), with excitation and emission wavelengths of 360 and 460 nm, respectively. The production of 4MU was linear with time for the incubation period used, and less than 10% of the available substrate was hydrolyzed. Experiments were performed in triplicate and spectra were corrected for the background emission from the buffer and the protein. Michaelis-Menten parameters were obtained by fitting the fluorescence intensity data with GraFit (Leatherbarrow, 2001).

IC_50_ determinations were carried out using substrate concentrations corresponding to the K_m_ established for *Cp*OGA (2.9 μM) and hOGA (80.0 μM). STZ was preincubated with the enzyme for 2 min to 18 hr. Gal-STZ was preincubated with the reaction mixture for 2 min.

Determination of the STZ K_i_ was performed by steady-state kinetics in the presence of different inhibitor concentrations (0, 30, 60, 120 μM). After 3 min preincubation of hOGA with STZ, the reaction was run for 60 min. The mode of inhibition was visually inspected by the Lineweaver-Burk plot, whereas K_i_s were determined by fitting all fluorescence intensity data to the standard equation for competitive inhibition in GraFit (Leatherbarrow, 2001).

### Protein Crystallography

*Cp*OGA crystals were produced as described previously (Rao et al., 2006). Precipitant was carefully removed and solid STZ was added straight to the drop. After 45 min, the crystal was removed and cryoprotected in mother liquor containing 15% glycerol. Diffraction data were collected to 2.2 Å on ID14-4 (European Synchrotron Radiation Facility), and processed with the HKL suite (Otwinowski and Minor, 1997), giving 99.8 % completeness, 3.6-fold redundancy, and an overall R_merge_ of 0.059. The structure was refined with REFMAC (Murshudov et al., 1997) together with model building in Coot (Emsley and Cowtan, 2004), giving a final model with good geometry (rmsd from ideal bonds = 0.012 Å; rmsd from ideal angles = 1.3°) and an R factor of 0.195 (R_free_ = 0.241).

### Synthesis of STZ and Gal-STZ

The procedures for preparation of *N*-methyl-*N*-nitrosocarbamic acid *N*′-hydroxysuccinimide ester and synthesis of STZ and Gal-STZ were adapted from Martinez et al. (1982). Synthesis of Gal-STZ is given as a representative procedure, as follows. To a stirred suspension of D-galactosamine hydrochloride 0.535 g (2.5 mmol) in methanol (10 ml), 25% stock sodium methylate solution in methanol (0.57 ml; 2.5 mmol) was added at room temperature to give a clear solution of free base. Then *N*-methyl-*N*-nitrosocarbamic acid *N*′-hydroxysuccinimide ester 0.553 g (2.75 mmol) was added in one portion to the above solution at 0°C (ice bath). The reaction was stirred for 10 min and then allowed to warm up to room temperature. At this point the solid went into solution. The reaction was cooled again to 0°C and further stirred for 3 hr. The reaction was evaporated to dryness. The residue was dissolved in dichloromethane:methanol (4:1) and quickly passed through a short pad of silica to remove unreacted galactosamine. The fractions containing the product were pooled and evaporated. The residue was dissolved in aqueous n-butanol (1:8; 15 ml). The solution was reduced to approximately one fourth of the initial volume when crystal deposition began. More n-butanol was added to complete sedimentation. The mixture was kept overnight at 4°C and filtered. Crystals were washed subsequently with ethyl acetate and ether and dried under vacuum to give 0.46 g (1.73 mmol, 69%) of the target product as pale yellow crystals. Melting point 144°C (decomposition); [α]_D_ = +77.8°; *c* 1.15 H_2_O. The twin set of signals in NMR spectra reflects the fact that Gal-STZ was obtained as a mixture of α:β anomers 1.6:1.

δ_H_ (500 MHz, D_2_O): 3.059 and 3.06 (3H, 2xs, CH_3_), 3.61 (0.6 H, dd, J_5,6a_ = 4.4 Hz, J_5,6b_ = 8 Hz, H-5β); 3.67 (3.2 H, m, H-6a,b; both isomers), 3.78 (0.6H, dd, J_3,2_ = 11 Hz, J_3,4_ = 3.3 Hz, H-3β), 3.87 (0.6H, d, H-4β), 3.94 m (2.6H, H-4α, H-3α, H-2β), 4.03 (1H, dd, J_5,6a_ = J_5,6b_ = 6.5 Hz, H-5α), 4.21 (1H, dd, J_2,1_ = 3.74 Hz, J_2,3_ = 10.8 Hz, H-2α), 4.7 (H-1β, obscured by water signal), 5.27 (1H, d, H-1α).

δ_C_ (125 MHz, D_2_O): 26.9 and 27 (CH_3_), 51.6 (2α), 55.1 (2β), 61 (6β), 61.2 (6α), 67.4 (3α), 68 (4β), 68.6 (4α), 70.6 (5α), 70.8 (3β), 75.2 (5β), 91.1 (1α), 95.2 (1β), 155.1, 155.5.

The stability of both STZ and Gal-STZ in aqueous solution was confirmed by NMR spectroscopy. No noticeable changes in ^1^H and ^13^C spectra were observed over a 16 hr period after dissolving STZ or Gal-STZ in D_2_O.

### Cell Culture

Mouse pancreatic Min6 insulinoma cells were a generous gift from Professor Jun-ichi Miyazaki, Osaka, Japan (Miyazaki et al., 1990). All tissue culture reagents were from Invitrogen. The cells were grown in a monolayer in Dulbecco's modified Eagle's medium supplemented with 15% fetal bovine serum at 37°C under 5% CO_2_. Gal-STZ and STZ were freshly prepared at the required concentration by dissolving them in prewarmed cell-culture medium. This Gal-STZ or STZ-containing medium was added to cells growing at a confluency of 50%–60% and incubated for the required amount of time depending on the experiment. A GlcNAcstatin stock (67 mM) was prepared in DMSO.

### Western Blotting

The anti-*O*-GlcNAc antibody CTD110.6 was purchased from Abcam. For western blotting, cells were lysed in lysis buffer containing 50 mM Tris-HCl (pH 7.5), 150 mM NaCl, 0.5% NP40 supplemented with protease inhibitor cocktail (Roche). Protein concentration was determined by Coomassie protein assay (Pierce). For immunoblotting, the protein samples were subjected to 10% SDS-PAGE, transferred to PVDF membrane, and blocked with 3% BSA before incubating with primary antibody and subsequently with conjugated anti-mouse IgM-HRP. To detect proteins, a chemiluminescent signal was developed using the ECL kit (Amersham Biosciences).

### DNA Fragmentation Assay

Min6 cells were grown in six-well plates and treated with 5–10 mM Gal-STZ or STZ or 20 μM GlcNAcstatin for 6 hr and then detached by trypsinization. A cell suspension of 4–6 × 10^5^ cells from each culture was pelleted at 2000 × g (5 min, 4°C) and subsequently lysed with 20 μl of lysis buffer (100 mM Tris-HCl [pH 8], 2 mM EDTA, 0.8% [w/v] SDS). RNA was removed by adding 2 μl of 50 mg/ml RNase A per sample, followed by incubating with 200 μg of proteinase K. After 2 hr incubation at 50°C, DNA loading buffer was added and the fragmented DNA samples were resolved on a 1.8% TBE-agarose gel, stained with SYBR gold (Molecular Probes), and scanned using a Fuji FLA-5000 with excitation at 493 nm and emission at 537 nm.

### Cell Viability and Annexin V-FITC Flow Cytometry

Min6 cells were grown in 24-well plates and treated with 5–10 mM Gal-STZ or STZ or 20 μM GlcNAcstatin, harvested after 6 hr, and stained with trypan blue to distinguish live from dead cells. An Annexin V-FITC (using the Annexin V-FITC apoptosis detection kit from BioVision) readout was used to quantitate cell viability through FACS. Min6 cells were plated at a density of 2 × 10^5^ cells and treated for 6 hr with 5–10 mM Gal-STZ or STZ or 20 μM GlcNAcstatin. After incubation, all the cells (attached and supernatant) were collected, washed with PBS, and resuspended in the Annexin V binding buffer and incubated with Annexin V for 5 min in the dark before analyzing with flow cytometry (ex = 488 nm; em = 530 nm) using a FITC signal detector (FL 1).

### Caspase 3 Activation Microscopy

To study Gal-STZ/STZ-induced apoptosis in fixed Min6 cells, a caspase 3 (Asp175) antibody (Cell Signaling Technology) was used which specifically detects the large fragment of the activated caspase 3. Min6 cells were grown on coverslips in six-well plates and treated with 5–10 mM Gal-STZ or STZ or 20 μM GlcNAcstatin for 8 hr. The cells were then fixed with 4% paraformaldehyde, blocked with 5% goat serum, incubated overnight with primary antibody, washed, and then incubated with a Texas red-conjugated anti-rabbit secondary antibody for 1 hr. After washing, the cells were mounted with Vectashield (Vector Labs) and examined under an LSM 510 Meta Zeiss microscope with an excitation wavelength of 543 nm (Texas red).

Live cell imaging was performed with a Leica DMIRB inverted microscope with a 10× phase contrast objective. Min6 cells were grown on an Ibidi microslide VI and 10 mM Gal-STZ or STZ was added to the cells, followed immediately by addition of 10 μM freshly prepared NucView 488 caspase 3 substrate (Biotium) and covered with mineral oil to reduce evaporation. The microslide was placed in an environmental chamber at 37°C to which the microscope was attached. The microscope, stage, and camera were controlled using Openlab (Improvision) software. The NucView 488 fluorescence signal was detected using a FITC filter set. The data were collected every hour for 12 hr from five different microscopic fields for each sample.

## Accession Numbers

Coordinates and structure factors of the *Cp*OGA-STZ complex have been deposited in the Protein Data Bank under ID code 2VUR.
